# Bursal Synovial Chondromatosis Secondary to Underlying Osteochondroma in a Child

**DOI:** 10.7759/cureus.1944

**Published:** 2017-12-14

**Authors:** Kumail Khandwala, Adeel A Waheed, Muhammad I Alvi, Waseem A Mirza, Masood Umer, Eraj Khurshid

**Affiliations:** 1 Department of Radiology, The Aga Khan University, Karachi.; 2 Department of Orthopaedic Surgery, The Aga Khan University, Karachi.

**Keywords:** synovial chondromatosis, osteochondroma, bone tumor, mri, bursa

## Abstract

Osteochondroma and synovial chondromatosis are frequently reported benign bony and cartilaginous lesions. Osteochondroma is distinguished by a cartilage-capped bony exostosis on the exterior surface of the bone, whereas synovial chondromatosis is secondary to metaplasia and is characterized by multiple cartilaginous loose bodies within the synovium. We present an atypical case of synovial chondromatosis developing in a bursa sec­ondary to an underlying osteochondroma of the proximal medial tibia in a child. It is extremely rare to see both these conditions occurring in one location simultaneously. Moreover, this association is an unusual occurrence in the pediatric age group. The patient underwent surgical excision of the lesions and the final diagnosis was confirmed on histology. Simulation to malignant degeneration is often observed and vigilant assessment of both lesions is essential to exclude the possibility of sarcomatous transformation in those who present with these conditions since clinical, radiological, and histopathological features may overlap. However, a proper preoperative distinction may prevent an unnecessary aggressive therapeutic approach, which stood true for our case.

## Introduction

Synovial chondromatosis in a bursa may develop as a result of chronic friction from an un­derlying osteochondroma. It is suggested that the osteochondroma causes an inflamma­tory reaction within the adjacent tenosynovium, which, over the passage of time, leads to chondromatous metaplastic transformation and formation of secondary synovial chondromatosis [1-3].

We present such a rare, histologically proven case of osteochondroma with secondary synovial chondromatosis simultaneously occurring in one location. This dual pathology is an unusual occurrence in the pediatric age group. Only a handful of case reports have documented this entity in adults, and, in most cases, the conditions were mimicking neoplastic degeneration of osteochondroma on imaging [4]. In instances where there is an abnormal appearance of a calcified mass overlying an osteochondroma, the diagnosis is most often peripheral chondrosarcoma, but the possibility of benign secondary synovial chondromatosis in an overlying bursa should also be kept in mind [4]. This case highlights some of the important distinguishing imaging features and reviews the previous literature on this association as well.

## Case presentation

**Figure 1:**
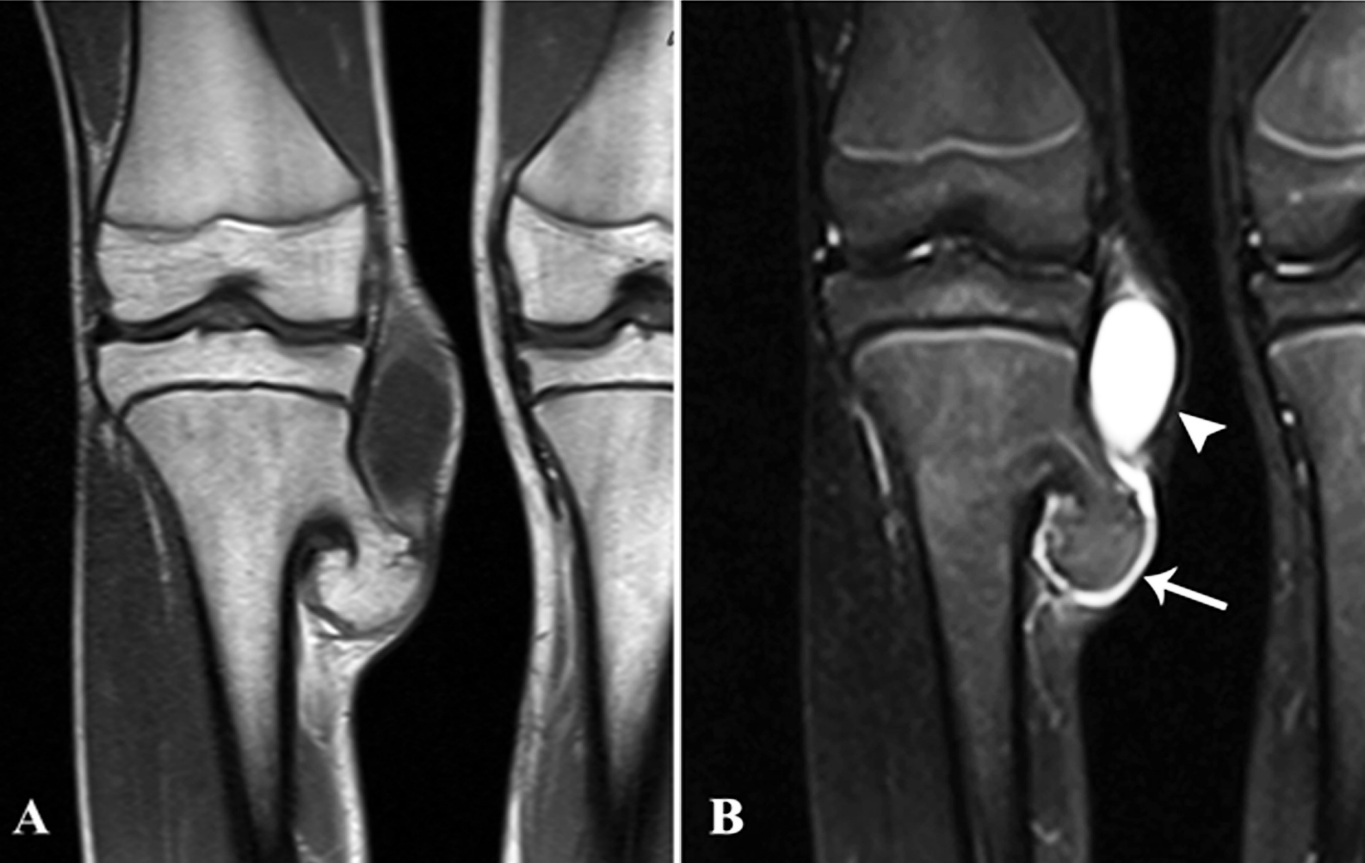
A & B: Coronal T1 post-contrast and STIR MR images Pedunculated bony protuberance from proximal medial tibial metaphysis demonstrating corticomedullary continuity with the bone and a thin cartilaginous cap (arrow). An adjacent fluid-filled, nonenhancing, partially covered bursa is also visualized on these sections (arrowhead). STIR: short TI inversion recovery; MR: magnetic resonance

A 15-year-old boy presented to our hospital with a bump over the medial aspect of the right leg for one year. The mass was painless at first, however, it started to increase in size, producing symptoms of discomfort for the last four months. His physical and systemic examination was otherwise unremarkable. Magnetic resonance imaging (MRI) was performed on clinical suspicion of a neoplastic mass. The scan revealed a pedunculated bony protuberance originating from the medial aspect of the proximal right tibia, measuring approximately 46 x 22 mm. It was demonstrating corticomedullary continuity with the underlying bone. The imaging features were consistent with an osteochondroma. A distinct cartilaginous cap was identified, which measured 2.5 mm in thickness (Figure 1).

**Figure 2:**
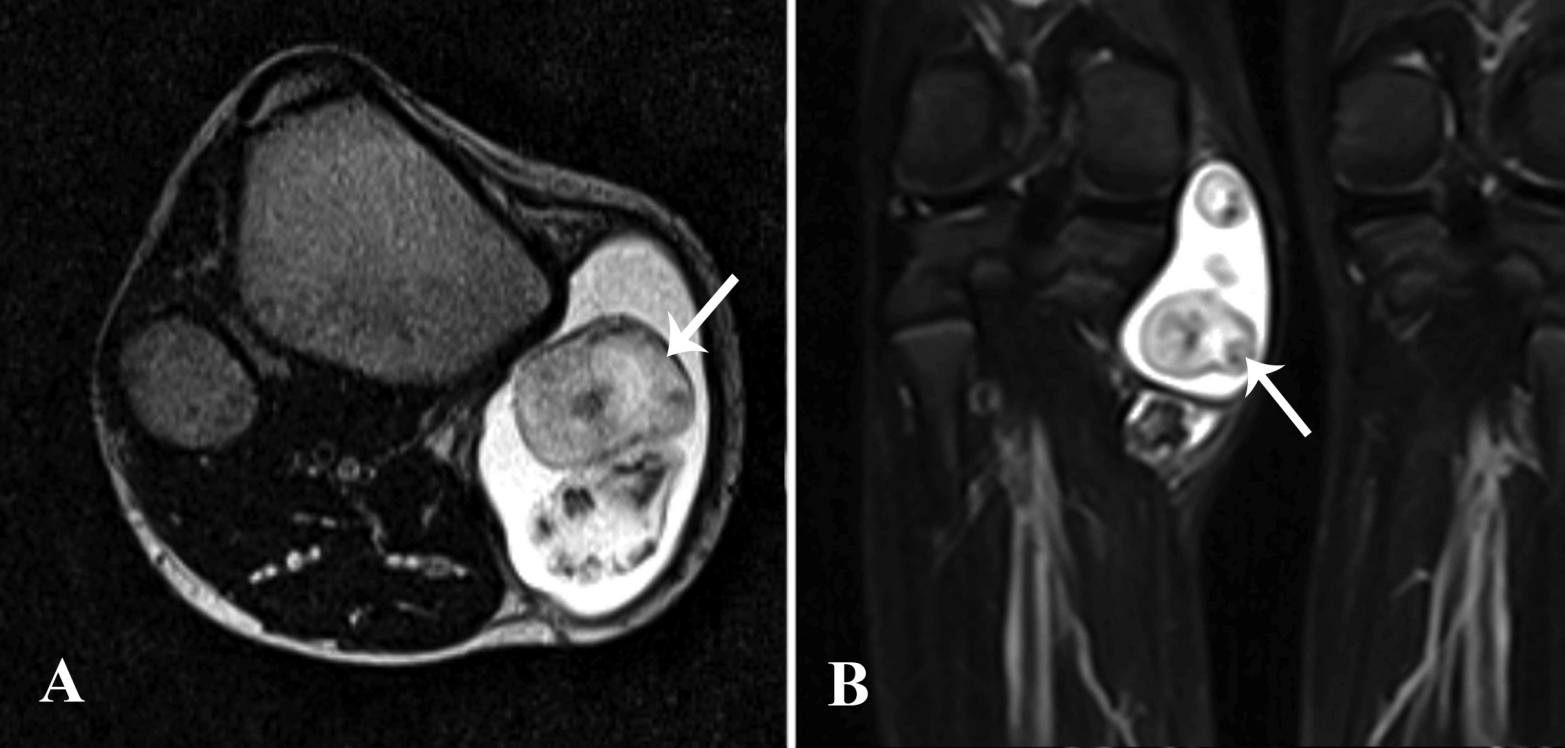
A & B: Axial T2-weighted and coronal STIR MR images Fluid-filled bursa in close proximity to the osteochondroma containing multiple, cartilaginous loose bodies (arrows) representing secondary synovial chondromatosis. STIR: short TI inversion recovery; MR: magnetic resonance

Additionally, there was a cystic fluid-filled structure adjacent to the cartilaginous cap, containing lobulated nodules suggestive of loose bodies. It measured 65 x 35 mm and was located superior to the osteochondroma at the medial aspect of the right knee joint (Figure 2). The findings were highly suggestive of synovial chondromatosis. On the basis of imaging features, there was no strong evidence to suggest sarcomatous transformation.

**Figure 3:**
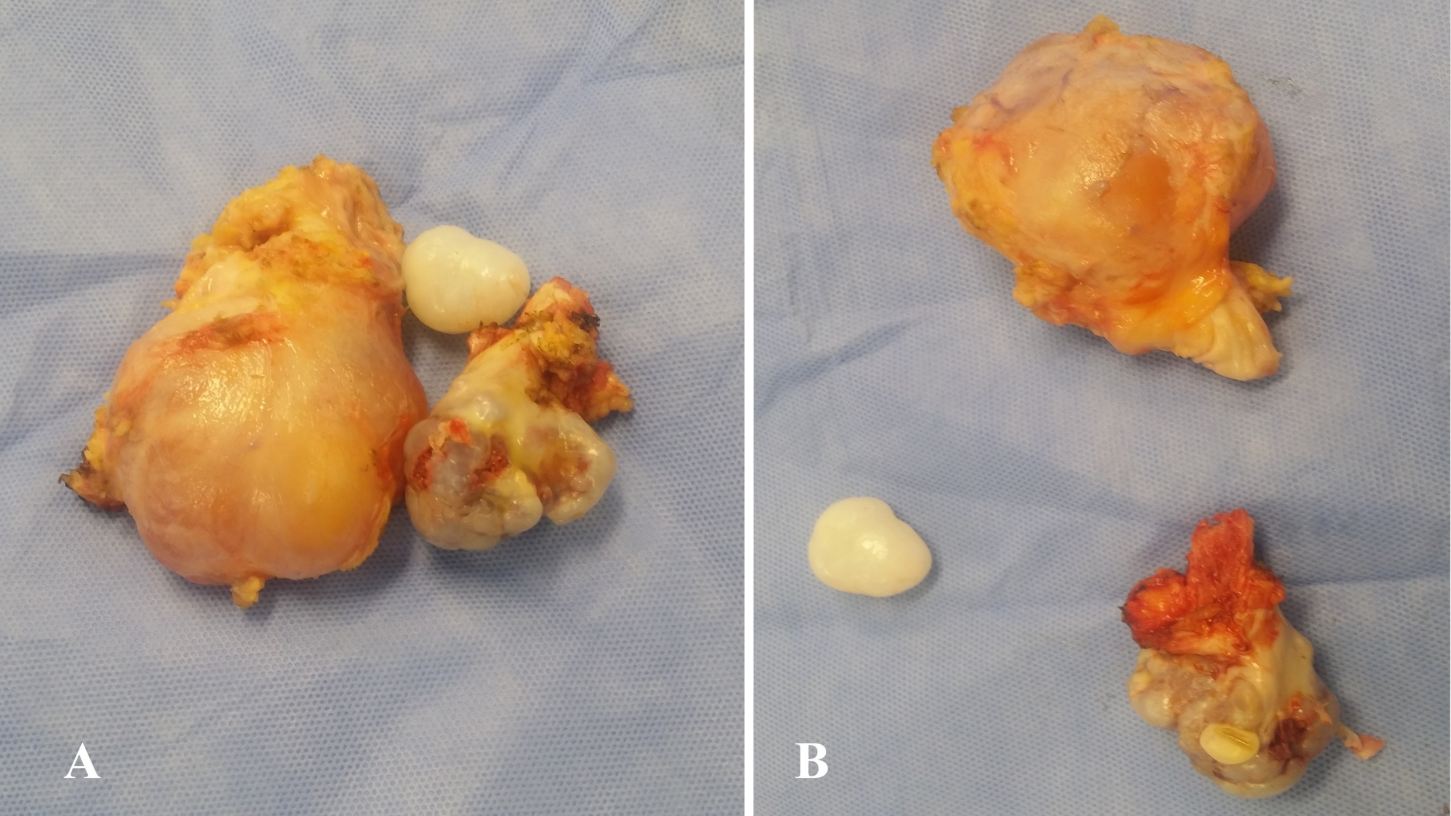
A & B: Operative specimens A) The excised osteochondroma and adjacent bursal synovial chondromatosis along with one of the cartilaginous loose bodies. B) The lesions separated out.

The patient underwent surgical excision of the above-mentioned lesions. Operative findings revealed a pedunculated bone lesion arising from the medial aspect of the proximal tibia and a cystic fluid-filled structure over the medial aspect of the knee, which was abutting but not involving the pes anserinus (conjoined tendon of the sartorius, gracilis, and semitendinosus muscles that inserts anteromedially on to the proximal tibia). Multiple cartilaginous loose bodies were found within the cystic sac (Figure 3).

Two separate surgical specimens were sent for histopathological anal­ysis. Sections examined from the bony lesion revealed normal bone with an outer layer that was the fibrous perichondrium and was continuous with the periosteum of the underlying bone. Within the cartilage cap, the chondrocytes were clustered. No evidence of a spindle cell component or atypia, necrosis, or mitosis was seen. Sections examined from cystic tissue revealed a cystic cavity lined with flattened cells with mild, nonspecific inflammation. It contained nodules of mature cartilage with a cut surface that was tan-white myxoid in appearance. The largest nodule measured 5 x 3 cm (Figure 3). There was no evidence of malignancy. The diagnosis was consistent with osteochondroma with synovial chondromatosis without sarcomatous transformation.

Post-operatively, the patient was initially kept on intravenous fluids, given analgesia, antibiotics, and antiemetics. He was eventually mobilized out of bed and physiotherapy was initiated. He started tolerating orally, showed good progress, and was discharged in a stable condition. He is presently doing well, has regained the full range of motion, and will be followed in the orthopedic clinic as an outpatient.

## Discussion

Osteochondromas are one of the most commonly reported bony lesions and constitute up to 20%-50% of all benign bone tumors. Osteochondromas are distinguished by a bony protuberance/exostosis surrounded by a cartilage cap. These exostoses are usually metaphyseal in location and occur most commonly in the immature skeleton (10-20-year age group). Osteochondromas are often asymptomatic but can present with a painless swelling, protru­sion, or bulge on the affected extremity. Two distinct clinical forms have been described, solitary or multiple osteochondromas [1].

Synovial chondromatosis is a condition in which there is a formation of osteochondral bodies in the synovial cavity due to metaplasia of the synovium. This condition mostly occurs in adults (4^th^ to 5^th^ decade of life) with a male preponderance. Synovial chondromatosis has been reported to occur in all synovial joints, including the temporomandibular joint. However, the knee joint is the most commonly affect­ed [2]. A metaplastic transformation of the synovium has been reported to follow an indolent course; however, the development of multiple, osteochondral loose bodies results in wear and tear of the articular cartilage, often leading to the development of secondary osteoarthritis [3].

Often, there is the formation of a bursa in areas where there is friction between the osteochondroma and overlying tissues, especially if they are large in size. Syno­vial chondromatosis rarely develops in these secondary reactive bursal or tenosy­novial spaces formed by primary under­lying osteochondromas [1,5-6]. The etiology of the formation of loose bodies in cases of underlying osteochondromas has always been subject to debate. One school of thought is that they are a result of dislodgement of lobulated nodules of osteochondroma by constant friction [1]. Another theory is that the growing osteochondroma causes synovial metaplasia, which leads to the formation of chondrocytes that then form calcified loose bodies that are sustained by the synovial fluid [3].

The clinical features of secondary synovial chondromatosis are difficult to differentiate from a solitary osteochondroma or primary synovial chondromatosis. Therefore, radiological imaging studies with computed tomography (CT) and MRI are vital to establish a diagnosis. On CT and MRI, osteochondroma characteristically shows continuity with the medullary cav­ity of the bone. On MRI, the cartilaginous cap shows an intermediate intensity on T1-weighted images and high signal intensity on T2-weighted images. Generally, it is thought that if the cartilaginous cap thickness is more than 2 cm on T2-weighted images, it is suspicious for malignant degeneration to peripheral chon­drosarcoma [6].

Classical imaging features of synovial chondromatosis include multiple intra-articular cartilaginous nodules/loose bodies with specific “ring-and-arc” chondroid mineralization and extrinsic erosion of bone on both sides of the joint [6]. The MRI has shown to be better for delineating calcified loose bodies as well [2].

Despite the above-mentioned features, a diagnosis based on clinical and radiographic findings may sometimes be challenging because synovial chondromatosis adjacent to osteochondroma can often simulate a peripheral chondrosarcoma. Between 1% and 5% of solitary osteochondromas have been reported to undergo a malignant transformation to the peripheral chondrosarcoma. Additionally, there have been special cases described in the previous literature in which transformation from synovial chondromatosis to chondrosarcoma has also occurred. The prevalence has been reported to be up to 5%. [7-8]. Osteochondroma with synovial chondromatosis has been previously observed in adults in which most of the cases were simulating neoplastic degeneration of osteochondroma [4,6]. This association was also seen in a 17-year-old male in the same anatomic location of the proximal tibia; however, we could not find any case report in the younger pediatric age group [9].

The imaging features were quite distinctive in our case, thus sparing the patient from a more aggressive therapeutic approach, leading to a shorter postoperative recovery time and faster regaining of the full range of motion. However, overlapping imaging features include an extension into the nearby soft tissues, especially into the bursa, and extrinsic erosion of bone. These may be seen in both synovial chondromatosis and chondrosarcoma, which is why it is sometimes difficult to distinguish between the two [8]. An MRI may adequately demonstrate the clear margin between the calcified mass and the underlying cartilage cap, as seen in our case. However, there are times when the loose bodies and fluid in the bursa can be mistaken for a thick cartilage cap seen in peripheral chondrosarcoma [7]. Previous reports have suggested that a permeative pattern with true cortical destruction and bone marrow infiltration should strongly raise the possibility of ma­lignancy. Therefore, the MRI has always been considered the gold standard for evaluating bone marrow involvement; thus, helping to distinguish it from deep ex­trinsic erosion, as observed in our case as well [6,8].

The true diagnosis is almost certainly based on the histology of the specimens. However, a pathological analysis of secondary synovial chondromatosis can sometimes lead to overdiagnosis of chondrosarcoma because there is a marginal difference between such benign cartilaginous lesions and low-grade chondrosarcoma [10]. Treatment strategies primarily include surgical resection. This has been shown to have a good response, with low chances of recurrence. In synovial chondromatosis, a radical resection of both loose bodies and the affected synovium should be performed. Local recurrence after complete resection, a rapid increase in size, a sudden increase in pain, or a deteriorating clinical course are worrisome signs of malignant transformation in patients with either of these lesions [9].

## Conclusions

Osteochondromas and synovial chondromatosis are common, benign bony and cartilaginous lesions; however, it is unusual to see both conditions in the same anatomic location simultaneously. This association is especially unusual in the pediatric age group, where synovial chondromatosis itself is rarely encountered. In instances where there is an abnormal calcified mass overlying an osteochondroma, the diagnosis is most often peripheral chondrosarcoma, but the possibility of benign synovial chondromatosis in an overlying bursa should always be considered in the differential diagnosis. Imaging modalities, such as CT and MRI, may help to establish the diagnosis; however, the final verdict is based on histopathology of the resected specimens.
